# Cost Analysis of the Belgian National Antimicrobial Resistance Monitoring in Livestock: Effects on Sampling Design and Statistical Performance

**DOI:** 10.3390/antibiotics15020172

**Published:** 2026-02-05

**Authors:** Maria Eleni Filippitzi, Adrien de Fraipont, Mickaël Cargnel, Céline Guillaume, Jean Baptiste Hanon

**Affiliations:** 1 Laboratory of Animal Production Economics, Faculty of Veterinary Medicine, Aristotle University of Thessaloniki, 54124 Thessaloniki, Greece; 2 Coordination of Veterinary Activities and Veterinary Epidemiology Service, Infectious Diseases in Animals Department, Sciensano, 1050 Brussels, Belgium; adrien.defraipont@sciensano.be (A.d.F.); mickael.cargnel@sciensano.be (M.C.); celine.guillaume@sciensano.be (C.G.); jean-baptiste.hanon@sciensano.be (J.B.H.)

**Keywords:** antimicrobial resistance (AMR), AMR monitoring, AMR surveillance, cost analysis, sample size, statistical analysis

## Abstract

Background/Objectives: As part of the European Union’s harmonized monitoring framework, Belgium conducts antimicrobial resistance (AMR) monitoring in commensal bacteria from livestock. The aim of this study was to conduct a cost analysis of the national AMR monitoring in livestock, and to explore sampling size scenarios in relation to their associated costs and statistical performance (power and confidence) of monitoring. Methods: To our knowledge, this is the first published cost evaluation using unit cost aggregation of a national AMR monitoring program in animals. Results: The testing of the different sample size scenarios showed that if the sample size increases, the costs increase linearly. A sample size increase of 10 samples/isolates (e.g., from 170 to 180) can increase the yearly total costs per animal species by 5.2%. Moreover, the testing of the different scenarios showed that if the sample size increases, the power and the confidence level also increase, providing a higher level of trust in the results of the monitoring program. The highest total monitoring costs per animal category were estimated for fattening pigs, broilers and veal calves (over 18% of total costs each, using 2024 data). Among the various monitoring activities, antimicrobial susceptibility testing emerged as the costliest component, representing 50.2% of the total monitoring costs. Conclusions: The approach presented allows it to be used by other countries aiming to estimate the cost of their national AMR monitoring in animals or other similar activities. This economic and scenario testing analysis can be used to suggest informed suggestions to improve AMR monitoring in animals.

## 1. Introduction

The antimicrobial resistance (AMR) monitoring of indicator bacteria in livestock animals is performed in Belgium according to the European Union (EU) requirements (2013/652/EU) and the European Food Safety Authority (EFSA) methodology, as described in the Report from the Task Force on Zoonoses Data Collection including guidance for harmonized monitoring and reporting of antimicrobial resistance in commensal *Escherichia coli*, *Enterococcus faecalis* and *Enterococcus faecium* [1,2,3]. The objective of these EU guidelines is to provide specifications for harmonized monitoring and reporting of antimicrobial resistance in *E. coli* and *Enterococcus* spp. from healthy food animals and to help Member States to provide antimicrobial resistance data that are comparable. The monitoring focuses on *E. coli* as a key indicator for Gram-negative bacteria and *E. faecium* and *E. faecalis* for Gram-positive bacteria. These bacteria act as reservoirs of resistance genes and reflect antimicrobial selective pressure in livestock.

As part of the EU’s harmonized monitoring framework, Belgium conducts AMR monitoring in commensal bacteria from livestock animals with the aim of tracking significant trends in resistance, assessing the impact of antimicrobial use when possible, and providing data for risk assessments and policy recommendations. In Belgium, *E. coli* monitoring has been conducted annually since 2011, while *Enterococcus* spp. monitoring took place annually, between 2011 and 2013, and after a pause was resumed in 2019. Sampling is primarily conducted at slaughterhouses by the Federal Agency for the Safety of the Food chain (FASFC), and targets broilers, fattening pigs and veal calves (cattle under one year old raised in large specialized fattening units). Additionally, young beef cattle (<7 months old, raised in beef cattle farms; only for *E. coli*), laying hens and breeding hens are monitored through farm sampling. *E. coli* monitoring in veal calves, broiler chickens, and fattening pigs is mandatory for EU member states, while for other species, monitoring is conducted by countries on a voluntary basis. *Enterococcus* monitoring is entirely voluntary for all species (CID 2020/1729).

The Belgian monitoring program aims to analyze at least 170 isolates per bacterial species per year (see Section 4.1). This allows the detection of a 15% change in widespread resistance prevalence (e.g., 50% resistance), a 5% increase in cases with low initial resistance levels (e.g., 0.1% resistance), and an 8% accuracy rate for estimating resistance proportions in a statistically worst-case scenario (50% resistant isolates). Fecal samples are taken by an official veterinarian or veterinary officer at the slaughterhouse or at the farm and transported under controlled conditions (0–4 °C) to the laboratories of the Federal Agency for the Safety of the Food chain (FASFC), where bacterial isolation is performed. AMR susceptibility testing is performed using phenotypic methods, including minimum inhibitory concentration (MIC) testing, at the AMR national reference laboratory of Sciensano, the Federal research institute for public and animal health in Belgium. Results from the AMR monitoring are published annually by Sciensano, e.g., [2,3,4].

The present analysis is part of a comprehensive evaluation study requested and funded by the Federal Public Service (FPS) of Health, Food Chain Safety and Environment and performed in 2024–2025 by Sciensano. The aim of this study was to evaluate thoroughly the AMR national monitoring program and, if deemed necessary based on the findings, to provide evidence-based recommendations to the competent authorities for its further optimization. This evaluation study included a SWOT analysis (to be published soon), which is a structured framework used to identify and evaluate the strengths, weaknesses, threats, and opportunities of the current AMR monitoring for *E. coli* and *Enterococcus* spp. in Belgian livestock. According to experts interviewed for the SWOT analysis, while they acknowledged several strengths of the current monitoring system, they also highlighted potential key weaknesses. For instance, concerns were raised as to whether the standard sample size (i.e., 170 isolates) is sufficient to detect meaningful changes in antimicrobial resistance trends. This issue is of particular relevance from a policy perspective, as resistance trends are often compared to national antibiotic usage data, e.g., [4], which is the focus of ongoing reduction efforts. More generally, the primary epidemiological purpose of the national AMR monitoring program is to quantify the prevalence of resistance and to track temporal trends in indicator and zoonotic bacteria from livestock, providing data that can be used to evaluate the evolution of resistance in the animal population and inform national and EU-level surveillance and policy frameworks. Such prevalence and trend data are also instrumental for interpreting associations with antimicrobial use and for One Health policy development. The aforementioned observations of the SWOT analysis raised questions about whether increasing the sample size would significantly raise monitoring costs, and to what extent this would impact the statistical power and confidence of the monitoring program.

To date, there has been no detailed cost analysis of the national AMR monitoring program in livestock in Belgium, and to our knowledge no comparable cost evaluations focusing specifically on national AMR surveillance in livestock have been published in the international literature. Systematic reviews of AMR economic evaluations report a broad lack of economic evidence on AMR interventions with almost all studies focused on human health settings and very few, if any, addressing livestock surveillance [5,6]. Methodological work on AMR surveillance economics exists in a conceptual or antimicrobial use (AMU) surveillance context [7]; however, empirical cost analyses of national AMR monitoring in livestock are absent or extremely limited. Therefore, the aim of this study—conducted as part of a broader evaluation activity to enhance the effectiveness and sustainability of the monitoring program—is to perform a cost analysis of the current national antimicrobial resistance monitoring system in livestock, and to explore different sampling size scenarios by explicitly balancing associated costs with statistical performance of the monitoring program. In particular, the study evaluates how different sample size configurations affect both the associated monitoring costs and the statistical performance of the system (i.e., power and confidence to detect meaningful AMR trends), with the objective of identifying an optimal trade-off between feasibility and epidemiological reliability.

## 2. Results

**Figure 1:**
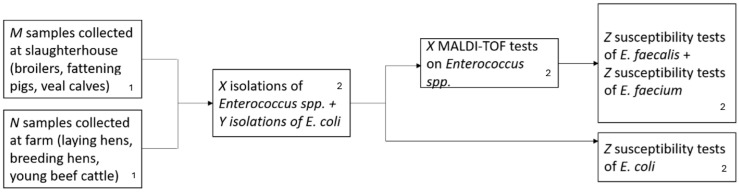
Scheme of the current process of activities (steps) of the AMR monitoring of indicator bacteria in livestock in Belgium. *N*, *M*, *X*, *Y*, *Z* refer to numbers. More details about these numbers can be found in Supplementary Materials (Table S1). ^1^ Activities FASFC official veterinarians or veterinary officers are responsible for. ^2^ Activities laboratories/Sciensano NRL AMR are responsible for.

Figure 1 illustrates the different steps (activities) of the monitoring process. These steps are: the (fecal) sampling at the slaughterhouse or at the farm level, depending on the animal category; the isolation of the bacteria of interest (i.e., *E. coli*, *E. faecalis* and *E. faecium*) from the collected fecal samples; the Matrix-assisted laser desorption/ionization (MALDI-TOF) testing, which is performed only for *E. faecalis* and *E. faecium* isolates to identify the bacteria species; and the susceptibility testing using Sensititre for the bacteria of interest (i.e., *E. coli*, *E. faecalis* and *E. faecium*).

### 2.1. Costs per Step of the Monitoring Process

The number of fecal samples collected, number of isolations, number of tests performed and costs (€) per activity (i.e., cost component) included in the AMR monitoring, for *E. coli*, *E. faecalis* and *E. faecium*, per livestock animal category included in the monitoring, per year are presented in Table S1.

#### 2.1.1. Sampling

The number of samples to be collected is determined based on the estimated isolation rate of the bacteria of interest in the fecal samples, to ensure a sufficient number of isolates for testing. Indeed, while this isolation rate is 100% for *E. coli*, for *Enterocci* it is expected to be less. For instance, for *E. faecalis* it has been estimated to be between 20% (breeding hens) and 69% (broilers), while for *E. faecium* between 46% (pigs) and 80% (laying hens) (2023 data) [8]. According to the current sampling strategy defined for 2025 (FASFC, pers. comm. M. Lebrun), the number of samples to be collected at the slaughterhouse level per year can be found in Table S1. It should be noted that the monitoring of AMR in young beef cattle is limited to *E. coli*, and also mentioned that, since the same samples are also used in the monitoring of AMR in zoonotic bacteria (mainly targets *Salmonella* in poultry), the current numbers are higher than what is needed for AMR monitoring in indicator bacteria. This practically means that not all samples collected will afterwards be isolated and tested for AMR in indicator bacteria. Thus, when considering monitoring scenarios that increase the number of isolates, the number of samples to be collected will not be modified up to a certain point.

According to the information provided about costs, the cost of sampling per one fecal sample collected at the slaughterhouse is estimated as *c*_1_ = 43.12 €/sample collected. At the farm level, the cost of sampling per one fecal sample collected is estimated as *c*_1_ = 65.59 €/sample collected. These estimations include labor and material costs. The total cost of sampling (at the farm or at the slaughterhouse level) per animal category per year (*C*_1_) (€) ranges from 11,609 € for young beef cattle to 14,561 to for laying hens and can be found in Table S1 for each category.

#### 2.1.2. Bacterial Isolation and Identification

According to the data provided by the FASFC, the number of isolations for *E. coli* and for *Enterococcus* spp. currently defined (data for 2025) can be found in Table S1.

The unit cost of bacterial isolation and identification is estimated as *c*_2*a*_ = 39 €/isolate for *E. coli* (source: FASFC, 2024, pers.comm. B. Pochet) and *c*_2*b*_ = 35.5 €/isolate for *Enterococcus* spp., which includes the cost of the MALDI-TOF to identify the species of *Enterococci* (source: standard prices of 2024 based upon convention between Sciensano and FASFC). The total cost of isolation and identification per animal category per year for both *E. coli* and *Enterococci* (*C*_2_) (€) ranges between 6903 € for young beef cattle and 16,542 € for broilers and fattening pigs and can be found in Table S1 for each category and for each bacterium separately.

#### 2.1.3. Susceptibility Testing

The number of susceptibility tests performed as part of the monitoring, together with the respective costs are presented in Table S1. Regarding the susceptibility tests, we consider that these are performed in a total target of 510 tests (170 tests for each of the three bacteria) for each livestock category participating in the AMR monitoring in Belgium. This is the case except for young beef cattle, who are submitted to a collection of 177 samples at the farm but are only used for *E. coli* detection, resulting in a total of 170 susceptibility tests.

Regarding the unit cost for susceptibility testing, it is communicated as *c*_4*a*_ = 58.58 €/test for *E. coli* (Sciensano, 2024, pers.comm.) and *c*_4*a*_ = 62.51 €/test for *Enterococcus* spp. (Sciensano, 2024, pers.comm.). In Belgium, the unit cost per susceptibility test is the same for *E. faecalis* and *E. faecium*. The total cost for susceptibility testing is the same for every animal category per year (*C*_4_ = 31,229 €) (except young beef cattle, as previously described), as the same number of tests are performed per bacteria per species per year (i.e., z = 170).

### 2.2. Total Cost, Power and Confidence Level of Monitoring for Different Sample Size Scenarios

The total cost of the AMR monitoring for animal category *i* per year (*C_Ti_*) and the total cost of the AMR monitoring for all animal species per year (*T*) for Belgium, using the available data, are presented in Table S1. It is observed that, with the exception of young beef calves (*C_T(Young_beef)_* = 28,488 €), the calculation of *C_Ti_* does not present large differences between the other species included in the monitoring, and ranges between 58,482 € (*C_T(Breeding_hens)_*) and 61,224 € (*C_T(Broiler_chickens)_* = *C_T(Fattening_pigs)_*). As for the total cost of the AMR monitoring for all animal species per year (*T*) calculated for Belgium given the available data, and considering susceptibility testing of 170 isolates for each of the three bacteria of interest (*E. coli*, *E. faecalis* and *E. faecium*), it is estimated as *T* = 330,655 €.

**Figure 2:**
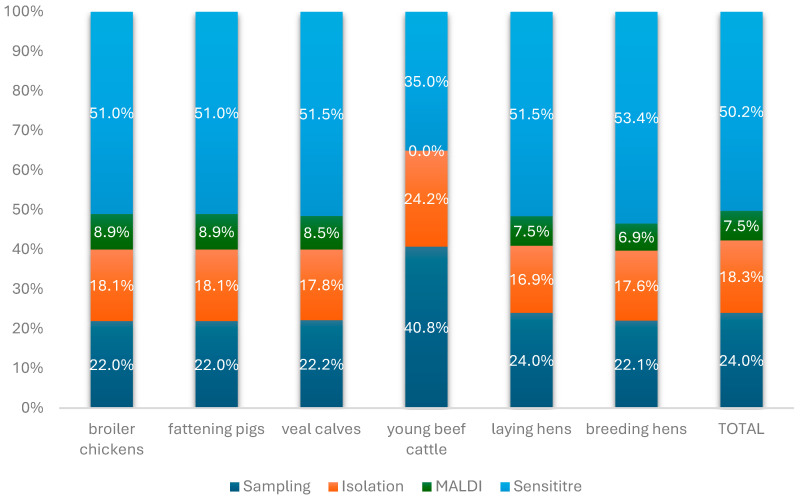
Proportional cost distribution of AMR monitoring stages across animal categories/species and overall, for all species together. Sampling refers to collection of fecal samples at slaughterhouse or farm. Isolation refers to laboratory isolation of bacterial isolates from fecal samples. MALDI refers to MALDI TOF testing for bacterial species identification. Sensititre refers to susceptibility testing of isolated strains.

Figure 2 illustrates the proportions of the monitoring cost allocated to the different cost components (steps) of the monitoring for each of the animal categories included and in total, in Belgium. Susceptibility testing appears as the major component of the monitoring cost for all animal categories (35–53%), followed by the samples collection (22–41%), the isolation of bacteria (17–24%), and finally the MALDI-TOF testing performed for identification of *Enterococci* (7–9%). The only exception are the young beef cattle, for which the collection of samples represents the biggest cost component of the monitoring program, followed by the susceptibility testing, and finally the bacterial isolation. This is explained by the fact that currently young beef cattle samples are only used for the monitoring of resistant *E. coli* and sampling at the farm is more expensive than at the slaughterhouse.

Table 1 summarizes the different estimated levels of the total cost of the AMR monitoring of the six studied animal categories per year (*T*) in Belgium, for the different scenarios of sample size, as well as the estimated power, confidence level and difference in prevalence possible to significatively detect according to EFSA treshold values (i.e., power of 80%, confidence of 95% and widesepread prevalence of 50%) for each of these scenarios. We can observe, indicatively (Table 1), that an increase of 10 samples, e.g., from 170 to 180, can lead to an increase in *T* of around 5.2%. Such an increase would also lead to an increase in power of around 2.7% (considering a confidence level of 95%), an increase in confidence level of around 0.9% (considering a power of 80%) and a decrease in the difference in prevalence of resistance that can be detected of 2.6% (considering a power of 80%, a confidence level of 95% and initial prevalence of 50%). Moreover, an increase of 25 samples, e.g., from 170 to 195, can lead to an increase in costs of around 12.9%, but also an increase in power of 6.2% and confidence of 1.9% and a decrease in the difference in resistance prevalence that can be detected of 6.3%.

**Figure 3:**
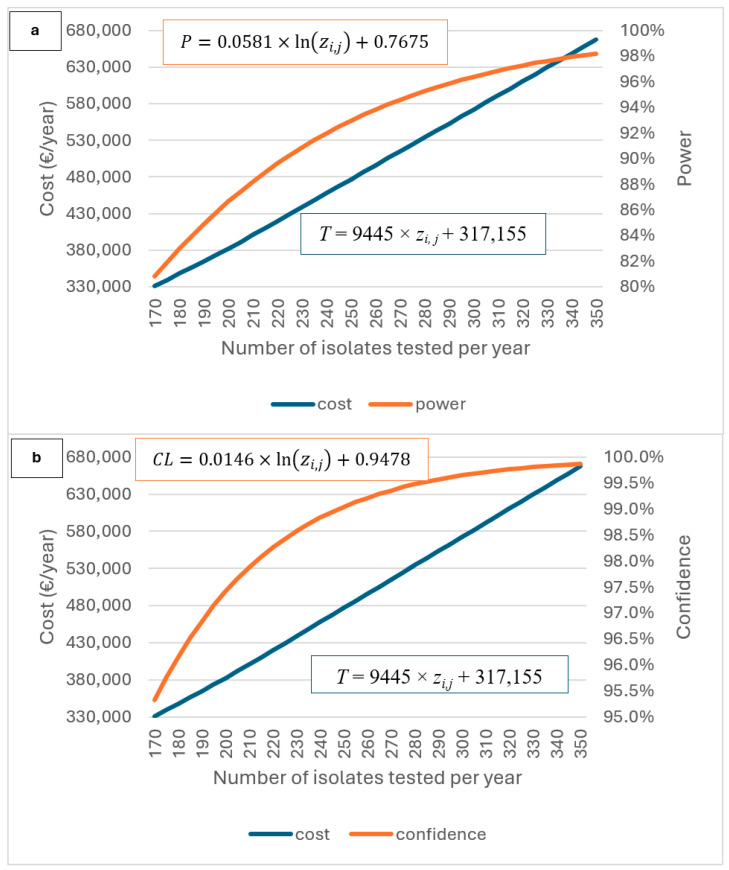
Graph showing the relationship between the sample size (i.e., number of isolates tested per year), the total cost of the monitoring (i.e., the six animal categories included) (*T*) and the power of monitoring (*P*), considering a 95% confidence level (*CL*) (**a**); and graph showing the relationship between the sample size (i.e., number of isolates tested per year), the total cost of the monitoring (i.e., the six animal categories included) (*T*) and the *CL*, considering a *P* of 80% (**b**). Based upon data from the Belgian AMR monitoring program. *z_i,j_* is the number of isolates tested per year and in the scenario analysis it is $170\leq z_{i,j}\;\leq 350$. Regression coefficient for *P* is R^2^ = 0.9618, for *T* is R^2^ = 0.9999 and for *CL* is R^2^ = 0.9843.

Figure 3a illustrates the effect that the increase in sample size can have on the total cost of the monitoring (i.e., the six animal categories included) (*T*) and on power, considering a 95% confidence level, while Figure 3b illustrates the effect that the increase in sample size can have on the total cost of monitoring (*T*) and on confidence level, considering a power of 80%. When fitting curves to the plotted estimated values of cost as function of sample size (Figure 3a), a linear relationship between the number of samples (i.e., the number of isolates tested) and the total cost (*T*) was observed:

*T* = 9445 × *z_i,j_* + 317,155; 170 ≤ *z_i,j_* ≤ 350, (1)

with a regression coefficient R^2^ = 0.9999, and where *T* is the total cost of the monitoring in €/year, and *z_i,j_* is the number of isolates tested per year.

Therefore, based on the linear approximation of the cost evolution curve, increasing on average the number of isolates tested (*z_i,j_*) by one additional isolate for all of six studied animal categories when 170 ≤ *z_i,j_* ≤ 350, would increase the total cost of the monitoring by 9445 €, using the Belgian data.

When fitting curves to the plotted estimated values of power of the monitoring (*P*) as function of number of isolates tested in Belgium, the curve observed in Figure 3a can be approached by the following logarithmic function:

(2) $$P=0.0581\times \ln\left(z_{i,j}\right)+0.7675;\;170\leq \;z_{i,j}\;\leq 350,$$

with a regression coefficient R^2^ = 0.9618, and where *z_i,j_* is the number of isolates tested per animal category i for bacteria j per year.

This indicates that, at a 95% confidence level, the power of monitoring increases along with sample size (i.e., number of isolates tested), but at a progressively slower rate. Such approximation is only valid in the defined interval of sample size value. In practice, at some point the power will tend toward the horizontal asymptote *p_a_* = 1. For instance, a power of 95% is reached once 276 isolates are tested for each animal category, and after this point the power increase becomes almost negligible.

When fitting curves to the plotted estimated values of confidence level (*CL*) as function of number of isolates tested in Belgium, the curve observed in Figure 3b can be approached by the following function:

(3) $$CL=0.0176\times \ln\left(z_{i,j}\right)+0.9478;\;170\leq z_{i,j}\;\leq 350,$$

with a regression coefficient R^2^ = 0.9843, and where *z_i,j_* is the number of isolates tested per year.

**Figure 4:**
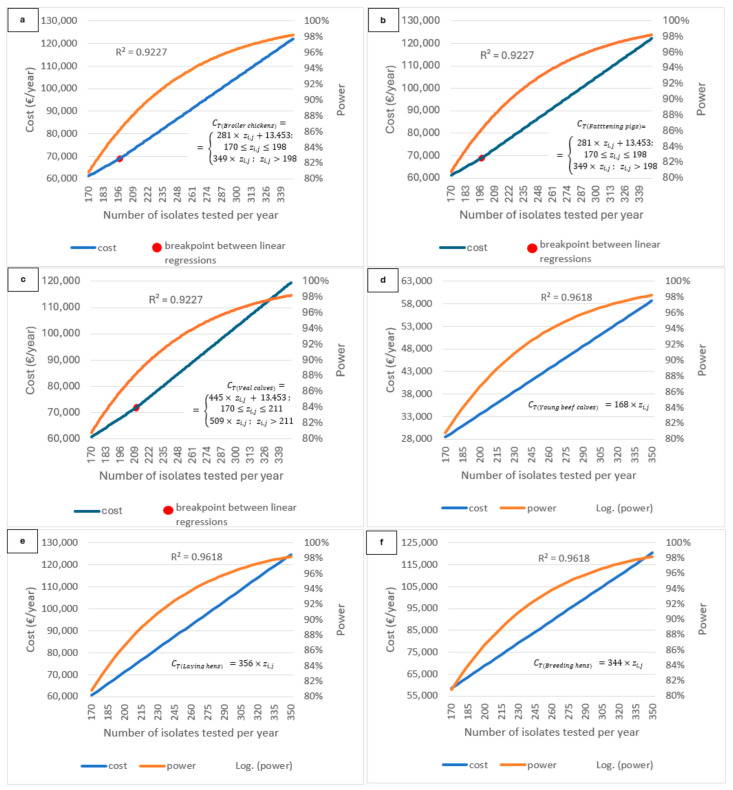
(**a**–**f**) Cost (*C_Ti_*) and power of the monitoring as a function of sample size (i.e., number of isolates tested) at a 95% confidence level for each animal category *i*, i.e., broiler chickens (**a**), fattening pigs (**b**), veal calves (**c**), young beef calves (**d**), laying hens (**e**) and breeding hens (**f**). For illustration purposes, we use data from Belgium. The formulas indicated on the graphs are the linear equations describing the cost (*C_Ti_*) evolution per animal category *i* as function of the sample size (i.e., number of isolates tested for the bacteria selected *j*). For broiler chickens, fattening pigs, and veal calves, the cost evolution as function of the sample size corresponds to two different linear regressions, with a switch point occurring at a specific sample size, highlighted in red in (**a**–**c**). As for *z_i,j_*, it is the number of isolates tested per year and in the scenario analysis it is $170\leq \;z_{i,j}\;\leq 350$.

Figure 4a–f show the effect that the increase in sample size (number of isolates tested) can have on the total cost of monitoring for each animal category per year (*C_Ti_*) and on power, considering a 95% confidence level. The cost evolution for each animal category (*C_Ti_*) as a function of the number of isolates tested for each of the bacteria of interest (*z_i,j_*) observed in Figure 4a–f are described by the following linear equations per animal category.

For broiler chickens and fattening pigs:

(4) $$C_{T(Broiler\;chickens)}=C_{T(Fattening\;pigs)}=\left\{\begin{array}{c} 281\times \;z_{i,j}\;+\;13,453\;;\;\;170\leq z_{i,j}\leq 198\\ 349\times \;z_{i,j}\;;\;\;z_{i,j}>198 \end{array}\right.,$$

For veal calves:

(5) $$C_{T(Veal\;calves)}=\left\{\begin{array}{c} 445\times \;z_{i,j}\;+\;13,453;\;\;170\leq z_{i,j}\leq 211\\ 509\times \;z_{i,j};\;\;z_{i,j}>211 \end{array}\right.,$$

For young beef calves:

(6) $$C_{T(Young\;beef\;calves)}=168\times z_{i,j},$$

For laying hens:

(7) $$C_{T(Laying\;hens)}=356\times z_{i,j},$$

For breeding hens:

(8) $$C_{T(Breeding\;hens)}=344\times z_{i,j},$$

It is observed that for three out of the six animal categories—the broiler chickens, fattening pigs, and veal calves—the cost evolution depending on sample size (i.e., number of isolates tested for each of the bacteria of interest) (Equations (4) and (5)) in Belgium corresponds to two different linear regressions, with a switch point occurring at a specific sample size, highlighted in red in Figure 4a–c. In detail, Equation (4) indicates that for broiler chickens and fattening pigs, increasing the sample size by one additional isolate results in a cost increase of 281 € up to a sample size of 198. Beyond this point, each additional sample increases the cost by 349 €. The equation also demonstrates that even if the sample size were reduced to zero—meaning no further monitoring of AMR in indicator bacteria—the total cost would still amount to 13,453 €. This residual cost corresponds to the collection of 312 fecal samples required for monitoring zoonotic bacteria.

Similarly, for veal calves, increasing the sample size by one additional isolate results in a cost increase of 445 € up to a sample size of 211. Beyond this point, each additional sample increases the cost by 509 €. Finally, for young beef cattle, each increase in sample size by one results in a cost increase of 168 €, for laying hens each increase in sample size by one results in a cost increase of 356 €, and for breeding hens each increase in sample size by one results in a cost increase of 344 €.

### 2.3. Sensitivity Analysis

**Figure 5:**
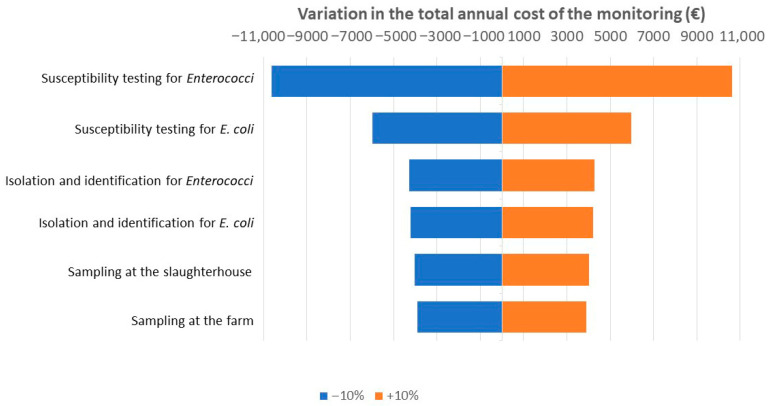
Sensitivity of the total annual cost of the monitoring to a ±10% variation in the various cost components.

The results of the sensitivity analysis are displayed in Table 2 and Figure 5. They indicate that the cost components having the highest impact on the output (i.e., the total annual cost of the monitoring) are in descending order: the susceptibility tests for *Enterococci*, the susceptibility tests for *E. coli*, the isolation and identification for *Enterococci*, the isolation and identification for *E. coli*, the sampling at the slaughterhouse, and the sampling at the farm.

Therefore, the total annual cost of the monitoring is mostly impacted by the cost of the bacterial susceptibility tests, especially for *Enterococcus*, meaning that variations in the cost of Sensititre, which is currently used, could lead to significant modification of the total cost. For instance, a 10% increase in this cost would result in an additional total cost of 10 627€ per year.

## 3. Discussion

To our knowledge, this is the first published cost evaluation of a national AMR monitoring program in animals. According to the estimations of the evaluation, in Belgium, the highest total monitoring costs per animal category were estimated for fattening pigs, broilers, and veal calves. Among the various monitoring activities, antimicrobial susceptibility testing emerged as the costliest component, representing approximately 35% (for young beef cattle) to 53.4% (for breeding hens) and 50.2% for all considered species of the total monitoring (*T*) expenditure.

The testing of the different sample size scenarios shows that if the sample size is increased, the costs also increase, linearly, as described in this exercise. For instance, according to our cost estimation model, an increase in the sample size by 10 samples, e.g., from 170 to 180, can lead to an increase in total monitoring costs per animal species per year of around 5.2% (Table 1). Moreover, the testing of the different sample size scenarios shows that if the sample size is increased, the power and the confidence level also increase, providing a higher level of trust in the results of the monitoring program (e.g., resistance prevalence) (Figure 3a,b and Figure 4a–f, as well as Table 1). In addition, the sample size (isolates to be tested) estimated according to the guidelines of EFSA is a minimum recommended one, leaving the freedom to the Member States to include more samples in their program, according to their goals and needs.

For instance, generally, when annual monitoring indicates an apparent increase or decrease in AMR prevalence for a specific antimicrobial compared with previous year(s), it may not always be possible to determine whether the observed change reflects a true shift in prevalence, when the difference between the two estimates is not statistically significant, due to imprecision arising from limited sample size (i.e., wide confidence intervals). In such cases, increasing the sample size in subsequent year(s)—even for a specific species–bacteria–antimicrobial combination—would improve the precision of prevalence estimates and increase statistical power, allowing true trends to be more reliably detected. Based upon the formula of EFSA [1] for the estimation of sample size, it is possible to determine the required size to show a significant increase or decrease in prevalence with a desired power.

Rather than identifying a single optimal scenario, the comparative analysis (Table 1) is intended to support informed decision-making by illustrating explicit cost–performance trade-offs relative to the EFSA-based baseline. While the baseline scenario already meets current regulatory recommendations, alternative scenarios demonstrate how increased investment translates into higher numbers of isolates and improved statistical precision. Conversely, lower-cost scenarios show the extent to which monitoring intensity can be reduced while maintaining acceptable performance levels. This framing allows program managers to select a scenario that best aligns with budgetary constraints and surveillance objectives, rather than prescribing a single preferred design.

While the EFSA technical specifications define minimum numbers of isolates to be tested per animal species and production category within the EU, these thresholds are primarily intended to ensure harmonization and baseline statistical power across Member States rather than to maximize sensitivity for the early detection of emerging resistance. As illustrated by our scenario analyses, reductions in sample size are expected to result in a proportional loss of statistical power and confidence, thereby increasing the risk of failing to detect low-prevalence or emerging resistance phenotypes. This relationship is not specific to Belgium and would similarly apply in monitoring systems inside or outside the EU, where sampling frameworks may be further constrained by financial or logistical limitations. In such contexts, reductions in the number of isolates tested—if not adequately compensated by targeted or risk-based sampling strategies—may substantially compromise both the sensitivity and representativeness of AMR systems. In addition to absolute sample size, the capacity of monitoring systems to detect emerging resistance is influenced by the geographic coverage and structural representativeness of sampling. Even when minimum isolate numbers are met, uneven spatial distribution or over-representation of specific herd types may limit extrapolation of results when scaling monitoring approaches to countries with different livestock production systems, herd densities, or diagnostic infrastructures.

The approach presented in this paper is developed in a way that allows it to be used by other countries aiming to estimate the cost of their national AMR monitoring in animals or other similar activities, using cost aggregation (i.e., summing disaggregated cost components across all surveillance activities). Such a method can be further incorporated in larger cost–benefit analyses. Regarding the actual calculations for Belgium, it should be noted that these are based on unit costs as provided by stakeholders involved in the monitoring program implementation (inter-agency communication), while some limited assumptions had to be used in some cases of parameter specification (e.g., use target numbers of fecal samples and isolates to be tested although slight variations might exist depending on year). Moreover, the calculations are linked to the current strategy and are subject to the economic situation of the specific time period. In case any change in unit costs and quantities arises, the output T can be easily calculated using this deterministic cost model, to reflect the new situation.

The cost analysis was based on programmatic financial data provided by the competent authorities in Belgium and reflects the real-world implementation of the national AMR monitoring program. The cost data include both variable (marginal) costs related to sampling and laboratory processing per isolate, as well as fixed operational costs associated with the routine functioning of the surveillance system. These fixed costs encompass logistics, personnel time, and laboratory infrastructure. However, the cost data were provided as aggregated annual totals rather than disaggregated by cost category. As a result, fixed costs were allocated proportionally across sampling scenarios to assess their impact on total program costs, while preserving consistency with the actual budgetary structure of the monitoring system. Although this approach limits further internal disaggregation, it ensures that the modeled scenarios remain grounded in observed expenditures and are directly relevant for decision-making within the current surveillance framework. We believe that explicitly documenting this aggregated approach aligns with common practice in public surveillance accounting and provides a practical and adaptable framework that other countries can use according to the level of cost detail available to them.

While the methodological framework is transferable, the absolute cost estimates generated are expected to vary substantially across countries due to differences in herd structure, baseline cost levels, labor costs, and laboratory organization. For example, countries with more centralized livestock sectors or higher degrees of laboratory automation may achieve lower marginal costs per isolate, whereas systems relying on decentralized sampling or manually intensive laboratory workflows may incur higher per-sample costs. Moreover, certain countries might include more species in their monitoring (e.g., EU Decisions 2013/652 and 2020/1729 require AMR testing on turkey caeca collected in slaughterhouses for the years 2022, 2024, and 2026, for national production exceeding 10,000 tons of turkey meat per year, like in Poland or Germany; not applicable to Belgium). Differences in diagnostic capacity—such as access to MALDI-TOF for bacterial identification or automated broth microdilution systems for antimicrobial susceptibility testing—may further influence both cost structures and data comparability. National surveillance programs such as DANMAP (Denmark) [9], MARAN (The Netherlands) [10], and NARMS (United States) [11] illustrate the variation in herd composition, sampling logistics, and laboratory capacity between countries. It could therefore be expected that this can lead to substantially different cost distributions across surveillance components. Consequently, the primary added value of the Belgian case study lies in the transparency and modularity of the costing approach rather than in the direct transferability of absolute cost figures.

Regarding the AMR monitoring in Belgium in broiler chickens, fattening pigs, and veal calves, using the entire set of fecal samples that are already being collected to isolate indicator bacteria and test them for AMR, could allow for obtaining a higher sample size at a lower cost. This is explained by the fact that the same samples collected for poultry and pigs are also used to monitor zoonotic bacteria (EC 2160/2003). Thus, more fecal samples are collected than what is needed to monitor resistant indicator bacteria, meaning that isolations and susceptibility tests of indicator bacteria are not performed currently on all the available samples (i.e., 312 samples are collected for broiler chickens, but only 268 isolates of *Enterococci* and 180 of *E. coli* are required, according to the current strategy). Therefore, it is possible to increase the number of isolates tested in the monitoring without having to increase the number of samples collected, up to a certain point. This leads to a cost evolution that presents a moderate acceleration once the number of isolations needed to obtain the appropriate targeted sample size exceeds the number of samples currently collected. However, since the costs linked to sampling account for 22% of the total monitoring costs in these three animal categories in Belgium, the total cost increase linked to a bigger sample size is mostly influenced by laboratory costs.

This economic and scenario testing (e.g., sample size—power) analysis is part of an evaluation exercise aiming to suggest informed suggestions to improve the current AMR monitoring in animals in Belgium. Other parts of the evaluation focused on the temporal and spatial representativity of sampling for the purpose of monitoring and the statistical method used to observe AMR trends. The economic analysis showed that increasing sample size will increase the cost of monitoring linearly. Therefore, any budget constraints will inevitably restrict the possibility to increase sample size significantly. Based on the results of this analysis and also taking into account the results of the other parts of the monitoring evaluation (Sciensano internal Scientific report to FPS), a number of respective recommendations can be made to improve monitoring without significantly increasing the total cost if possible. First, an adaptation (increase) of the sample size (i.e., number of isolates tested) could be made, in case a trend in AMR of a specific pathogen–antibiotic–animal category is observed, to increase statistical power and capture any potential change in prevalence of the respective specific resistance level. This is particularly interesting in some cases (e.g., of low AMR prevalence), where 170 isolates may not be enough to detect statistically significant increases/decreases while a quick estimation of the AMR trend is needed (e.g., for critical antimicrobials). Furthermore, a case-by-case decision could be made, for some livestock categories where AMR is less critical and observed AMR prevalences are lower (e.g., beef cattle), to reduce the frequency of sampling from annually—as currently performed—to biannually—as recommended by EFSA. Instead, the spared budget could be dedicated to an increase in the sample size (to increase power) or to monitor another livestock category/species (e.g., dairy cattle), while the total cost of the program would not be increased. Finally, this detailed cost analysis of the AMR monitoring program of Belgian livestock allows to identify the breakdown of costs for each step of the monitoring by animal category/species and can be a useful source of information for the authorities to manage the available total budget. Indeed, it can be used to make strategic decisions such as allocating resources to the monitoring of another animal species, changing the frequency of testing (annually/biannually) or improving efficiency in the implementation of some components of the process.

## 4. Materials and Methods

To achieve the aim of this study, we collected detailed data of costs linked to the different steps of the monitoring of antimicrobial resistance in animals in Belgium, from the different partners involved in it. This data has been provided by FASFC and Sciensano that lead and/or participate in the different monitoring activities and have access to this data (inter-agency communication).

### 4.1. Estimation of Costs per Step of the Monitoring Process

This section summarizes the method of the estimation of costs per each step/activity of the AMR monitoring, according to the information collected and concerns the sampling, the isolation, the identification, and susceptibility testing processes. First, information was collected from FASFC and Sciensano about: the number of samples collected at the slaughterhouse level or at the farm level for the different animal categories of the monitoring; the number of isolations and identifications performed per type of bacteria for the different animal categories; and the number of susceptibility tests performed per type of bacteria for the different animal categories (Table S1). The cost of MALDI-TOF used to identify species of *Enterococci* is included in the “isolation and identification” cost. This information is also referred to as ‘quantities’ (Table S1). Then, this information, together with the data on costs (also referred to as ‘unit costs’) per step of the monitoring collected (also referred to as ‘cost component’), was used by following unit cost aggregation, e.g., [12], to estimate the total annual cost of monitoring per animal category. Table 3 summarizes the type of information considered for this estimation, as well as the formulas used.

Regarding the number of samples, it should be noted that sample size in this study refers to the number of bacterial isolates that are aimed at being tested for resistance, and not to the number of swabs (fecal samples) that are aimed at being collected at slaughterhouses and farms. Although the recommended sample size (i.e., in the sense of isolates to be tested) according to the EFSA guidelines is a minimum one (167), Belgium is already including more samples (i.e., isolates) in the monitoring (170 isolates for *E. coli*, plus an increase in fecal samples for *Enterococci* until the number of isolates that can be tested for *Enterococci* is reached). Regarding the animal categories included, turkeys were not included in this analysis, as they are seldomly monitored on a voluntary basis since Belgian turkey production is below the threshold for own production monitoring. For example, over the past six years, they were only included in the 2022 monitoring program.

#### 4.1.1. Sampling

The fecal samples are collected either at the slaughterhouse or at the farm, and the same samples are used to monitor various bacterial species, both indicator and zoonotic. The samples collected at the slaughterhouse concern the monitoring of AMR for broiler chickens, fattening pigs and veal calves, while the samples collected at the farm concern the monitoring of AMR for laying hens, breeding hens and young beef cattle. For the purposes of this study, the respective numbers of samples actually collected at the two different levels and per animal category were provided by the FASFC and refer to the current sampling strategy defined for 2025. It should be noted that there may be some variations in the number of fecal samples collected depending on the year, as it results from the successful isolation rate of *Enterococci* observed in the previous year.

Regarding the cost per sample, for the farm level, we were provided with data of the total budget foreseen for labor and materials for the sampling for five years [13]. For the slaughterhouse level, we were provided with data of the total labor cost used in 2023, for broilers and fattening pigs (source: FASFC, 2023 data, pers.comm.). Therefore, this data was also used as proxy for each of the following years, and for labor cost for veal calves. As for the material costs, it was hypothesized that they equal those at the farm.

To estimate the cost of sampling (at the farm or at the slaughterhouse level) for an animal category *i* per year (*C*_1*i*_) (€), the following formula was used:

(9) $$C_{1i}=n_{i}*c_{1},$$

where *n_i_* is the number of samples collected per animal category per year and *c*_1_ is the cost of sampling per one sample (€). The cost of sampling is the sum of the cost of labor and the cost of materials.

#### 4.1.2. Bacterial Isolation and Identification

In order to obtain 170 isolates for susceptibility testing from the collected fecal samples, a certain number of these are used to isolate and identify the bacteria of interest, depending on their estimated prevalence. The quantities currently defined and the respective unit cost for the isolation of *E. coli* and *Enterococci* (*E. faecalis* and *E. faecium*) for each animal category included in the AMR monitoring in Belgium were provided by the FASFC (2025 data, pers.comm.). It is to be noted that samples from young beef cattle are used for *E. coli* detection only.

To estimate the cost of isolation for the bacteria of interest for the AMR monitoring *j* (i.e., *E. coli*, *E. faecalis* and *E. faecium*) for an animal category *i* per year (*C*_2*i*_) (€), the following formula can be used:

(10) $$C_{2i}=\;\sum_{j=1}^{n}\;\;x_{i,j}\;*\;c_{2j},$$

where *x_i,j_* is the number of isolations per animal category *i* for bacterium *j* per year and *c*_2*j*_ is the unit cost per isolation for bacterium *j* (€). In Belgium, the data about the quantity and unit cost of isolations for *E. faecalis* and *E. faecium* were provided as one total for both types of *Enterococci* included in the AMR monitoring.

#### 4.1.3. Susceptibility Testing

Regarding the susceptibility tests, we consider that these are performed on each of the 170 (target number) *E. coli*, 170 *E. faecalis*, and 170 *E. faecium* isolates obtained for each animal category, according to legislation and voluntary initiative, as previously described. Even though rarely and upon availability of budget, a few more isolates are tested (e.g., 178 *E. coli* from broilers in 2023), to achieve the aim of this study (i.e., compare costs in various scenarios), we considered for our estimations the “basis” scenario of 170 susceptibility tests. Samples from young beef cattle are used for *E. coli* detection only and, thus, do not undergo isolation of *Enterococci* or any further testing for *Enterococci*. The tests are performed for bacterial species identification according to the procedures of the laboratories, and only isolates meeting the predefined identification score thresholds were retained for further analysis.

For the cost of susceptibility testing for the bacteria of interest for the AMR monitoring (i.e., *E. coli*, *E. faecalis* and *E. faecium*) for an animal category per year (*C*_3*i*_) (€), it can be calculated by using the following formula:

(11) $$C_{3i}=\;\sum_{j=1}^{n}\;\;z_{i,j}\;*\;c_{3j},$$

where *z_i,j_* is the number of susceptibility tests per animal category *i* for bacterium *j* per year and *c*_3*j*_ is the unit cost per susceptibility test for bacterium *j* (€).

### 4.2. Estimation of Total Cost, Power and Confidence Level of Monitoring for Different Sample Size Scenarios

The annual total cost of the AMR monitoring per animal category *i* (*C_Ti_*) can be calculated by using a unit cost aggregation approach (Table 3). The respective formula is the following:

(12) $$C_{Ti}=\;C_{1i}+C_{2i}+\;C_{3i},$$

where $C_{1i}$ is the cost of sampling (at the farm or at the slaughterhouse level) for an animal category per year, calculated using Equation (9); $C_{2i}$ is the cost of isolation for the bacteria of interest for the AMR monitoring (i.e., *E. coli*, *E. faecalis* and *E. faecium*) for an animal category per year, calculated using Equation (10); $C_{3i}$ is the cost of susceptibility testing for the bacteria of interest for the AMR monitoring (i.e., *E. coli*, *E. faecalis* and *E. faecium*) for an animal category per year, calculated using Equation (11). The calculations for the estimation of the annual total cost of the AMR monitoring per animal category, based on the data collection performed as part of the study (see Section 4.1.1, Section 4.1.2 and Section 4.1.3), were performed in Excel.

The total cost for the AMR monitoring of all animal species per year (*T*) (€) is estimated by summing the annual total cost of the AMR monitoring per animal category (*C_Ti_*):

(13) $$T=\;\sum_{i=1}^{n}\;\;C_{Ti},$$

where *C_Ti_* is the annual total cost of the AMR monitoring per animal category *i*, estimated using Equation (12).

Following, a prospective analysis was performed to evaluate the trade-offs between sample size, statistical power, confidence level, and total cost for the proposed monitoring program. The power of monitoring refers to its ability to detect a significant variation in the prevalence of resistant indicator bacteria should one exist. For instance, power of 80% indicates that there is a 20% probability of failing to detect such change (i.e., false negative result). The confidence level reflects the confidence with which monitoring can detect a significant variation in the AMR prevalence. For example, a 95% confidence level indicates a 5% probability of incorrectly detecting such change occurring (i.e., false positive result). It should also be highlighted that, in this exercise (i.e., scenario testing of different sample sizes), the term “sample sizes” refers to the number of isolates on which susceptibility testing is performed (i.e., currently 170 isolates is the target), and does not refer to the number of fecal samples collected in the beginning of the monitoring process, which depends on the animal category.

For the purposes of this analysis, the power of the monitoring program and the confidence level of it were calculated for different scenarios of sample sizes. A range of sample sizes (i.e., *n*_min_ = 170, which is the minimum suggested sample size after rounding the recommended by EFSA value of 167, to *n*_max_ = 350) was considered. For each sample size, we estimated the following:

The annual total cost of the AMR monitoring per animal category *i* (*C_Ti_*) was calculated using Equation (5) and the total cost of the monitoring (*T*) using Equation (6) above. The possible total costs were estimated for every scenario between *n*_min_ and *n*_max_, with a difference of 5 samples considered from each previous scenario (see Table 1 for *T*).

The power (*P*) and the confidence level (*CL*) of the AMR monitoring were calculated for each animal category and for all animal categories per year, for the various scenarios of sample sizes, using the following formulas [1,14]:

(14) $$P=\;\varphi \left(\sqrt{\frac{n}{21.22}}-\varphi^{-1}\left(\frac{1+C}{2}\right)\right),$$

(15) $$CL=2\times \varphi \left(\sqrt{\frac{n}{21.22}}-\varphi^{-1}\left(P\right)\right)-1,$$

where *P* represents the power of the AMR monitoring, *CL* the confidence level of the AMR monitoring, *n* the annually required sample size, *φ* the cumulative distribution function of the standard normal distribution and *φ*^−1^ its inverse (See Table 3). When estimating *P* for a specific sample size, we considered *CL* = 95%, while when estimating the *CL* for a specific sample size, we considered *P*= 80%, similar to the values of the current program.

Following this, we plotted the estimated power and cost as functions of sample size and fitted curves to these data. The best-fitting models were selected based on visual fit and goodness-of-fit statistics (i.e., R^2^).

In addition, the difference in prevalence of resistance between years that can be significantly detected by the monitoring process was also calculated for increasing values of sample tested isolates. To do so, the references used to define a significantly detected difference were the threshold values defined by EFSA [1], meaning a power of 80%, a confidence of 95%, and a widespread scenario of 50% prevalence. Then, the following formula, which is derived from the sample size formula [1,14], was used:

(16) $$\Delta p=0.5\times \sqrt{\frac{15.68}{n+7.84}}$$

where *Δp* represents the difference in prevalence that can be significantly detected by the AMR monitoring and *n* the annually required sample size (See Table 3).

### 4.3. Sensitivity Analysis

A sensitivity analysis was conducted to assess the impact of potential variability in the estimated total monitoring costs. Such variability may arise from uncertainty in cost estimation or from temporal fluctuations. A ±10% variation was applied individually to the following input parameters of the model: the sampling costs at the farm and slaughterhouse, the isolation and identification costs for *Enterococcus* spp. and *E. coli*, and the costs of antimicrobial susceptibility testing for *Enterococcus* spp. and *E. coli.* Each input parameter was varied independently, first decreased to 90% of its baseline value (lower bound) and then increased to 110% (upper bound), while all other parameters were held constant. For each variation, the model output—defined as the total monitoring cost—was recalculated, allowing the relative influence of each cost component on the total cost estimate to be assessed.

## 5. Conclusions

This study presents an economic analysis of national antimicrobial resistance monitoring in livestock based on cost aggregation, using Belgium as a case study. The results highlight clear cost–performance trade-offs, with increasing sample sizes leading to higher costs alongside improvements in statistical power and confidence. In the Belgian context, monitoring costs were highest for fattening pigs, broilers, and veal calves, while antimicrobial susceptibility testing was identified as the main cost driver; these patterns may differ in other national settings. By explicitly quantifying these relationships, the analysis supports transparent, evidence-based decisions on monitoring design. The proposed framework is transferable and can inform the optimization of AMR monitoring and surveillance programs across different operational contexts and resource settings.

## Figures and Tables

**Table 1:** Summary of the different estimated levels of total cost of the AMR monitoring per year (*T*), for different scenarios of sample size, as well as the estimated power (*P*) and confidence level (*CL*) and difference in resistance prevalence that can be detected for each of these scenarios.

Isolates Tested ^1^ per Bacterium per Year	Total Cost of the Monitoring (*T*) (€/Year)	Power (*P*) for *CL* = 95%	Confidence Level (*CL*) for *P* = 80%	Difference in Resistance Prevalence That Can Be Detected (*Δp*) for *CL* = 95%, *P* = 80%, Initial Prevalence = 50%
170 ^2^	330,655	80.80%	95.33%	14.85%
175	339,193	81.91%	95.77%	14.64%
180	347,731	82.96%	96.16%	14.45%
185	356,270	83.96%	96.52%	14.26%
190	364,808	84.90%	96.85%	14.08%
195	373,346	85.80%	97.15%	13.90%
200	382,168	86.65%	97.41%	13.73%
205	391,386	87.46%	97.66%	13.57%
210	400,604	88.22%	97.88%	13.41%
215	410,111	88.94%	98.08%	13.26%
220	419,648	89.61%	98.26%	13.12%
225	429,186	90.26%	98.42%	12.98%
230	438,723	90.86%	98.57%	12.84%
235	448,260	91.43%	98.71%	12.71%
240	457,798	91.97%	98.83%	12.58%
245	467,335	92.48%	98.94%	12.45%
250	476,873	92.95%	99.04%	12.33%
255	486,410	93.40%	99.13%	12.21%
260	495,948	93.83%	99.22%	12.10%
265	505,485	94.22%	99.29%	11.99%
270	515,023	94.60%	99.36%	11.88%
275	524,560	94.95%	99.42%	11.77%
280	534,098	95.28%	99.47%	11.67%
285	543,635	95.59%	99.52%	11.57%
290	553,172	95.88%	99.57%	11.47%
295	562,710	96.15%	99.61%	11.38%
300	572,247	96.41%	99.65%	11.28%
305	581,785	96.65%	99.68%	11.19%
310	591,322	96.87%	99.71%	11.11%
315	600,860	97.08%	99.74%	11.02%
320	610,397	97.28%	99.76%	10.93%
325	619,935	97.46%	99.79%	10.85%
330	629,472	97.63%	99.81%	10.77%
335	639,010	97.80%	99.83%	10.69%
340	648,547	97.95%	99.84%	10.62%
345	658,084	98.09%	99.86%	10.54%
350	667,622	98.22%	99.87%	10.47%

Notes: ^1^ In other words, this is the number of susceptibility tests performed (*z_i,j_*). ^2^ This is the current scenario (2025) in Belgium.

**Table 2:** Results of the sensitivity analysis, indicating the variations in the total annual cost of the monitoring estimated for a ±10% variation in the various cost components of this process.

Cost Component	Total Annual Cost of the Monitoring Estimated		
	−10%	Initial Estimation	+10%
Sampling at the farm	326,746 €	330,655 €	334,564 €
Sampling at the slaughterhouse	326,619 €	330,655 €	334,691 €
Isolation and identification for *Enterococci*	326,367 €	330,655 €	334,944 €
Isolation and identification for *E. coli*	326,435 €	330,655 €	334,875 €
Susceptibility testing for * Enterococci *	320,029 €	330,655 €	341,282 €
Susceptibility testing for *E. coli*	324,670 €	330,655 €	336,641 €

**Table 3:** Summary of variables (in this case, types of quantities and types of unit costs) and formulas used to estimate the total annual cost of the monitoring per animal category and for all categories.

Variable	Description	Value or Formula	Source of Information
*n_i_*	Number of samples collected per animal category *i* per year	See Table S1, per species *i*	FASFC, 2025 ^1^
*c*_1_	Unit cost per sampling	See Table S1	FASFC, 2023 ^1^; [13]
*C*_1*i*_	Cost of sampling (at the farm or at the slaughterhouse level) for an animal category *i* per year	$C_{1i}=n_{i}\;*\;c_{1}$	
*x_i,j_*	Number of isolations and identifications per animal category *i* for bacterium *j* per year	See Table S1, per species *i*	FASFC, 2025
*c*_2*j*_	Unit cost per isolation and identification for bacterium *j*	See Table S1	FASFC, 2024 ^1^; Sciensano, 2024 ^1^
*C*_2*i*_	Cost of isolation and identification for the bacteria of interest for the AMR monitoring *j* (i.e., *E. coli*, *E. faecalis* and *E. faecium*) for an animal category *i* per year	$C_{2i}=\sum_{j=1}^{n}\;\;x_{i,j}\;*\;c_{2j}$	
*z_i,j_*	Number of susceptibility tests per animal category *i* for bacterium *j* per year	See Table S1, per species *i*	FASFC, 2025
*c*_3*j*_	Unit cost per susceptibility test for bacterium *j*	See Table S1	Sciensano, 2024
*C*_3*i*_	Cost of susceptibility testing for the bacteria of interest for the AMR monitoring (i.e., *E. coli*, *E. faecalis* and *E. faecium*) for an animal category per year	$C_{3i}=\sum_{j=1}^{n}\;\;z_{i,j}\;*\;c_{3j}\;\;$	
*C_Ti_*	Total cost of the AMR monitoring per animal category *i* per year	*C_Ti_* = $C_{1i}+C_{2i}+\;C_{3i}$	
*T*	Total cost of the AMR monitoring of all animal species per year	$T=\sum_{i=1}^{n}\;\;C_{Ti}$	
*φ*	Cumulative distribution function of the standard normal distribution	$\varphi (x)=\frac{1}{\sqrt{2\times \pi }}\times \int_{-\infty }^{x}e^{\frac{{-t}^{2}}{2}}dt$	
*P*	Power of the AMR monitoring	$P=\varphi \left(\sqrt{\frac{n}{21.22}}-\varphi^{-1}\left(\frac{1+C}{2}\right)\right)$	[1,14]
*CL*	Confidence level of the AMR monitoring	$CL=2\times \varphi \left(\sqrt{\frac{n}{21.22}}-\varphi^{-1}\left(P\right)\right)-1$	[1,14]
*Δ_p_*	Difference in resistance prevalence that can be significantly detected by the AMR monitoring	$\Delta p=0.5\times \sqrt{\frac{15.68}{n+7.84}}$	[1,14]

Notes: ^1^ Inter-agency communication.

## Data Availability

The raw data supporting the conclusions of this article are available as presented by the authors and/or the people from the institutions that provided them (see Acknowledgments).

## Supplementary Materials

The following supporting information can be downloaded at: https://www.mdpi.com/article/10.3390/antibiotics15020172/s1, Table S1. Summary of quantity (number) and unit cost (€) for each cost component (step/activity) of the AMR monitoring in livestock in Belgium, for *E. coli*, *E. faecalis* and *E. faecium*, and total cost of the monitoring (€) per livestock animal category included in the monitoring, per year.

## Abbreviations

The following abbreviations are used in this manuscript:

AMR	Antimicrobial resistance
EFSA	European Food Safety Authority
EU	European Union
FASFC	Federal Agency for the Safety of the Food chain
MIC	Minimum inhibitory concentration
