# Supplementary material for: Cost Analysis of the Belgian National Antimicrobial Resistance Monitoring in Livestock: Effects on Sampling Design and Statistical Performance
Source: Antibiotics (Basel). 2026 Feb 5;15(2):172. doi: 10.3390/antibiotics15020172 (PMC12937450; doi:10.3390/antibiotics15020172)
Supplement: Supplementary file 1 [file antibiotics-15-00172-s001.zip › antibiotics-4076837-supplementary.pdf]

Table S1. Summary of quantity (number) and unit cost (€) for each cost component (step/activity) of the AMR monitoring in livestock in Belgium, for *E. coli*, *E. faecalis* and *E. faecium,* and total cost of the monitoring (€) per livestock animal category included in the monitoring, per year.

Animal category	Cost component [1]	Unit sample	Unit cost (€)	Quantity	Total cost (€)
**Broiler chickens**	Sampling at the slaughterhouse	Sample	43.12	312	13 453
	Isolation and identification for *E. coli*	Isolation	39	180	7 020
	Isolation and identification for *Enterococci* [2]	Isolation	35.53	268	9 522
	Susceptibility testing for *E. coli*	Test	58.68	170	9 976
	Susceptibility testing for *Enterococci*	Test	62.51	340	21 253
	**Total cost [3]**				**61 224**
**Fattening pigs**	Sampling at the slaughterhouse	Sample	43.12	312	13 453
	Isolation and identification for *E. coli*	Isolation	39	180	7 020
	Isolation and identification for *Enterococci* [2]	Isolation	35.53	268	9 522
	Susceptibility testing for *E. coli*	Test	58.68	170	9 976
	Susceptibility testing for *Enterococci*	Test	62.51	340	21 253
	**Total cost [3]**				**61 224**
**Veal calves**	Sampling at the slaughterhouse	Sample	43.12	312	13 453
	Isolation and identification for *E. coli*	Isolation	39	180	7 020
	Isolation and identification for *Enterococci* [2]	Isolation	35.53	252	8 954
	Susceptibility testing for *E. coli*	Test	58.68	170	9 976
	Susceptibility testing for *Enterococci*	Test	62.51	340	21 253
	**Total cost [3]**				**60 656**
**Laying hens**	Sampling at the farm	Sample	65.59	222	14 561
	Isolation and identification for *E. coli*	Isolation	39	177	6 903
	Isolation and identification for *Enterococci* [2]	Isolation	35.53	222	7 888
	Susceptibility testing for *E. coli*	Test	58.68	170	9 976
	Susceptibility testing for *Enterococci*	Test	62.51	340	21 253
	**Total cost [3]**				**60 581**
**Breeding hens**	Sampling at the farm	Sample	65.59	197	12 921
	Isolation and identification for *E. coli*	Isolation	39	188	7 332
	Isolation and identification for *Enterococci* [2]	Isolation	35.53	197	7 000
	Susceptibility testing for *E. coli*	Test	58.68	170	9 976
	Susceptibility testing for *Enterococci*	Test	62.51	340	21 253
	**Total cost [3]**				**58 482**
**Young beef cattle [4]**	Sampling at the farm	Sample	65.59	177	11 609
	Isolation and identification for *E. coli*	Isolation	39	177	6 903
	Susceptibility testing for *E. coli*	Test	58.68	170	9 976
	**Total cost [3]**				**28 488**
**All animal categories**	**Total cost [3]**				**330 655 [5]**

Notes: 1. Corresponds to each step (activity) of the AMR monitoring, 2. Refers to *E. faecalis* and *E. faecium,* 3. Refers to total cost of AMR monitoring per year, 4. Samples from young beef cattle are only used for *E. coli* detection, 5. Refers to total cost for the AMR monitoring of all animal species per year (*T*), considering susceptibility testing of 170 isolates for each of the three bacteria of interest.
